# Intestinal Short Chain Fatty Acids and their Link with Diet and Human Health

**DOI:** 10.3389/fmicb.2016.00185

**Published:** 2016-02-17

**Authors:** David Ríos-Covián, Patricia Ruas-Madiedo, Abelardo Margolles, Miguel Gueimonde, Clara G. de los Reyes-Gavilán, Nuria Salazar

**Affiliations:** Probiotics and Prebiotics Group, Department of Biochemistry and Microbiology of Dairy Products, Instituto de Productos Lácteos de Asturias, Consejo Superior de Investigaciones CientíficasVillaviciosa, Spain

**Keywords:** short chain fatty acids, diet, human health, intestinal microbiota, cross feeding

## Abstract

The colon is inhabited by a dense population of microorganisms, the so-called “gut microbiota,” able to ferment carbohydrates and proteins that escape absorption in the small intestine during digestion. This microbiota produces a wide range of metabolites, including short chain fatty acids (SCFA). These compounds are absorbed in the large bowel and are defined as 1-6 carbon volatile fatty acids which can present straight or branched-chain conformation. Their production is influenced by the pattern of food intake and diet-mediated changes in the gut microbiota. SCFA have distinct physiological effects: they contribute to shaping the gut environment, influence the physiology of the colon, they can be used as energy sources by host cells and the intestinal microbiota and they also participate in different host-signaling mechanisms. We summarize the current knowledge about the production of SCFA, including bacterial cross-feedings interactions, and the biological properties of these metabolites with impact on the human health.

## Introduction

The gut microbiota influences our health and nutritional stage via multiple mechanisms, and a mounting body of evidence recognizes that microbial metabolites have a major influence on host physiology. Short chain fatty acids (SCFA) are volatile fatty acids produced by the gut microbiota in the large bowel as fermentation products from food components that are unabsorbed/undigested in the small intestine; they are characterized by containing fewer than six carbons, existing in straight, and branched-chain conformation. Acetic acid (C2), propionic acid (C3), and butyric acid (C4) are the most abundant, representing 90–95% of the SCFA present in the colon. The main sources of SCFA are carbohydrates (CHO) but amino acids valine, leucine, and isoleucine obtained from protein breakdown can be converted into isobutyrate, isovalerate, and 2-methyl butyrate, known as branched-chain SCFA (BSCFA), which contribute very little (5%) to total SCFA production. The aim of the present mini-review is to summarize the current knowledge about SCFA production, including bacterial cross-feedings interactions, and the biological properties of these metabolites with impact in human health.

## Mechanisms of SCFA production

### Metabolic routes

The main end products resulting from the CHO catabolism of intestinal microbes are acetate, propionate, and butyrate. Lactate, although is not a SCFA, is also produced by some members of the microbiota, such as lactic acid bacteria, bifidobacteria, and proteobacteria, but under normal physiological conditions it does not accumulate in the colon due to the presence of some species, such as *Eubacterium hallii*, that can convert lactate into different SCFA (Flint et al., 2015).

**Figure 1:**
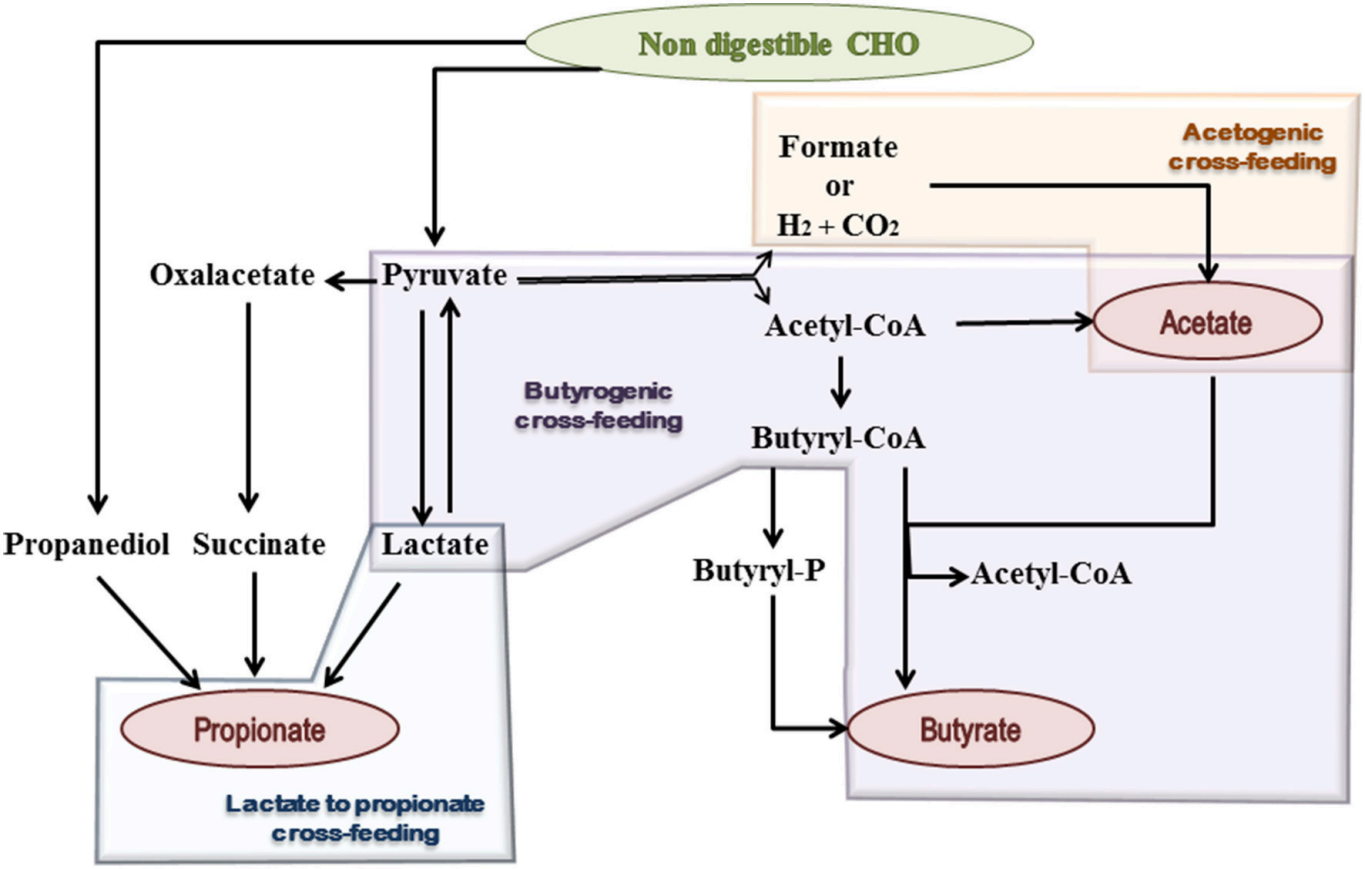
**Schematic representation of microbial metabolic pathways and cross-feeding mechanisms, contributing to SCFA formation in the human gut**. Shaded geometric shapes summarize routes of formation for each of the three main SCFA: acetate, propionate, and butyrate.

Acetate is the most abundant SCFA in the colon and makes up more than half of the total SCFA detected in feces (Louis et al., 2007). Two main metabolic routes have been described for acetate production by the gut microbiota (Figure 1). The majority of acetate is produced by most enteric bacteria as a result of CHO fermentation. In addition, approximately one-third of the colonic acetate is coming from acetogenic bacteria, which are able to synthesize it from hydrogen and carbon dioxide or formic acid through the Wood–Ljungdahl pathway (Miller and Wolin, 1996; Louis et al., 2014).

Propionate and butyrate metabolism have received much attention during the last years, mainly due to the connection between low levels of butyrate and propionate bacterial producers and some diseases in which inflammatory processes are involved. For instance, butyrate producers are normally low in ulcerative colitis (Machiels et al., 2014) and reduced levels of propionate producers have been detected in children at risk of asthma (Arrieta et al., 2015).

Three different pathways are used by colonic bacteria for propionate formation: succinate pathway, acrylate pathway, and propanodiol pathway (Reichardt et al., 2014) (Figure 1). The succinate route utilizes succinate as a substrate for propionate formation and involves the descarboxylation of methylmalonyl-CoA to propionyl-CoA. This pathway is present in several *Firmicutes*, belonging to the *Negativicutes* class, and in *Bacteroidetes*. In the acrylate pathway lactate is converted to propionate through the activity of the lactoyl-CoA dehydratase and downstream enzymatic reactions; this route appears to be limited to a few members of the families *Veillonellaceae* and *Lachnospiraceae* (Flint et al., 2015). In the propanodiol pathway, characterized by the conversion of deoxy-sugars to propionate, the CoA-dependent propionaldehyde dehydrogenase, that converts propionaldehyde to propionyl-CoA, has been suggested as a marker for this route. This metabolic pathway is present in bacteria which are phylogenetically distant, including proteobacteria and members of the *Lachnospiraceae* family (Louis et al., 2014; Reichardt et al., 2014). The relative abundance of *Bacteroidetes* has also been linked to the total fecal propionate concentration, suggesting that the succinate pathway is the dominant route within the gut microbiota (Salonen et al., 2014).

Two different pathways for butyrate production are known in butyrate-producing bacteria (Figure 1). The butyrate kinase pathway employs phosphotransbutyrylase and butyrate kinase enzymes to convert butyryl-CoA into butyrate (Louis et al., 2004). This route is not common among members of the gut microbiota and is mainly limited to some *Coprococcus* species (Flint et al., 2015). In contrast, the butyryl-CoA: acetate CoA-transferase pathway, in which butyryl-CoA is converted to butyrate in a single step enzymatic reaction, is used by the majority of gut butyrate-producers (Louis et al., 2010), including some of the most abundant genera of the intestinal microbiota, such as *Faecalibacterium, Eubacterium*, and *Roseburia*. Remarkably, the production of butyrate and propionate by the same bacterium is not common and only a few anaerobes, such as *Roseburia inulinivorans* and *Coprococcus catus*, are able to produce both (Louis et al., 2014).

### Cross-feeding mechanisms

Bacterial cross-feeding has a huge impact on the final balance of SCFA production and the efficient exploitation of the substrates that reach the human gut. These mechanisms consist either in the utilization of end products from the metabolism of a given microorganism by another one, called metabolic cross-feeding (Figure 1), and/or the utilization by one microorganism of the energy rich complex CHO breakdown products formed by another one, called substrate cross-feeding (Belenguer et al., 2006; Flint et al., 2007). A recent *in silico* study showed that mutualism cross-feeding interactions were promoted by anoxic conditions, which are more common in the large intestine than in the small one (Heinken and Thiele, 2015).

Microorganisms that are not capable of using complex CHO may proliferate by taking advantage of substrate cross-feeding, using breakdown compounds produced by hydrolytic bacteria. This is the case of some *Bifidobacterium* species that are not able to use inulin-type fructans (ITF) but can grow by cross-feeding of mono- and oligosaccharides released by primary inulin degraders in fecal cultures added with inulin as carbon source (Rossi et al., 2005; Salazar et al., 2009). Other example is the degradation of agaro-oligosaccharides (AO), which is more effective when *Bacteroides uniformis* and *Escherichia coli* are grown in co-culture than in separated monoculture (Li et al., 2014). In the same study the authors suggest the utilization of agarotriose, an intermediate in the degradation of AO, by *Bifidobacterium adolescentis* and *Bifidobacterium infantis*. In another work, it was demonstrated that *Roseburia* sp. strain A2-183 is unable to use lactate as carbon source, but when it is co-cultured with *B. adolescentis* L2-32 in the presence of FOS or starch, produces butyrate (Belenguer et al., 2006).

Although, there are a lot of *in vitro* studies pointing to metabolic cross-feeding it was not until recently that was demonstrated *in vivo* by using stable isotopes of acetate, propionate and butyrate perfused into the caecum of mice (Den Besten et al., 2013a). This study evidenced that the bacterial cross-feeding occurred mainly from acetate to butyrate, at lower extent between butyrate and propionate, and almost no metabolic flux exists between propionate and acetate. *In vitro* utilization of acetate by *Faecalibacterium prausnitzii* and *Roseburia sp*. has been evidenced (Duncan et al., 2002, 2004b). Prediction of metabolic fluxes between *F. prausnitzii* A2-165 and *B. adolescentis* L2-32 in co-culture has been reported in a computational model (El-Semman et al., 2014) and was recently demonstrated *in vitro* (Rios-Covián et al., 2015). Moreover, a recent animal study suggests that *F. prausnitzii* is able to use the acetate produced by *Bacteroides thetaiotaomicron in vivo*, this interaction having a significant impact in the modulation of the intestinal mucus barrier (Wrzosek et al., 2013). Although lactate is not a SCFA, it is used by some butyrate and propionate producing bacteria, avoiding metabolic acidosis in the host (El Aidy et al., 2013). Several *in vitro* studies confirm that lactate and/or acetate produced by *Bifidobacterium* when grown in oligofructose, is used by members of *Roseburia, Eubacterium*, and *Anaeroestipes* genera (Duncan et al., 2004a,b; Belenguer et al., 2006; Falony et al., 2006). Members of *Veillonella* and *Propionibacterium* are capable of transforming lactate to propionate *in vitro* (Counotte et al., 1981). H_2_ plays an important role in cross-feeding as well. Co-cultures of *Roseburia intestinalis* with the methanogen *Methanobrevibacter smithii* and the acetogen *Blautia hydrogenotrophica*, resulted in a decrease of final H_2_ and the production of CH_4_ and acetate. The acetate formed is used by *R. intestinalis* to produce butyrate (Chassard and Bernalier-Donadille, 2006). *Ba. thetaiotaomicron* bi-associated mice with *Bl. hydrogenotrophica* showed higher levels of acetate in caecal contents and lower NADH/NAD^+^ ratio; the removal of H_2_ by *B. hydrogenotrophyca* in this case allows *Ba. thetaoitaomicron* to regenerate NAD+ (Rey et al., 2010).

## Impact of diet on gut microbiota composition and SCFA production

Diet affects the gut microbiota composition and activity, and therefore the profile of SCFA and BSCFA synthesized, this having a deep impact on human health (Brussow and Parkinson, 2014; Louis et al., 2014). The first work linking the long-term diet style with the so-called human “enterotypes” was published in 2011 (Wu et al., 2011) but it has been also demonstrated that short-term diets can alter the human gut microbiome (David et al., 2014). The amount and relative abundance of SCFA may be considered as biomarkers of a healthy status (Table 1A). For example, high fiber-low fat and meat diets are characterized by the presence of higher amounts of fecal SCFA than diets with reduced fiber intake (De Filippo et al., 2010; Cuervo et al., 2013; Ou et al., 2013). A reduction in fecal butyrate has been found in patients with colorectal adenocarcinoma (Chen et al., 2013), whereas obesity has been related with increases in total fecal SCFA concentration (Fernandes et al., 2014; Rahat-Rozenbloom et al., 2014) which tend to decrease following an anti-obesity treatment (Patil et al., 2012). These epidemiological data have been further supported by dietary intervention studies carried out with different human populations (Table 1B). Prebiotic substrates that selectively promote the growth of beneficial microbiota also induce changes in SCFA production of healthy individuals (Lecerf et al., 2012) and in patients with irritable bowel syndrome or those receiving enteral nutrition (Majid et al., 2011; Halmos et al., 2015). Interestingly, the consumption of dairy products fermented with beneficial bacteria also modifies the intestinal microbiota toward more butyrate producers in comparison to chemically-acidified milk (Veiga et al., 2014). Finally, dietary intervention studies carried out with different overweight and obese populations seemed to be effective in lowering the high levels of fecal SCFA associated with the obesity status (Salazar et al., 2015).

**Table 1:** **(A) Epidemiological studies, carried out since 2010, showing the impact of diet on SCFA produced by the gut microbiota. The shaded areas indicate a change in the populations analyzed in terms of their health status. D, days; y, year. (B) Intervention studies, carried out since 2010, showing the impact of diet on SCFA produced by the gut microbiota. The shaded areas indicate a change in the populations analyzed in terms of their health status. D, day; w, week; m, month; y, year**.

**(A)**			
**Subjects, age (*****n*****)**	**Parameters determined**	**Main results**	**References**
•European children, 1–6 y (15) •Burkina Faso (BF) (rural) children (15)	3-d dietary questionnaire (from EU parents) and interview on diet (from BF mothers), fecal samples	**BF children**: ↑SCFA;↑*Bacteroidetes*, ↓*Firmicutes*, ↓*Enterobacteriaceae*; unique *Prevotella, Xylanibacter* (lacking in EU)	De Filippo et al., 2010
•Healthy African Americans, 50–65 y (12) •Healthy South Africans (12)	Fresh fecal samples, microbiota and SCFA analysis, cancer biomarkers	**Native Africans**: ↑SCFA, total bacteria, major butyrate-producing groups, dominance of *Prevotella* **African-Americans**: dominance of *Bacteroides*	Ou et al., 2013
•Healthy elderly, 76–95 (32)	Food frequency questionnaire, fecal SCFA analysis	**Correlation fiber and SCFA**: Potato intake with total SCFA and apple with propionate	Cuervo et al., 2013
•Overweight (OWO) (11) •Lean (11)	3-d diet record, fresh fecal sample, SCFA absorption measure	**OWO**: ↑Age-adjusted fecal SCFA concentration, not due to higher absorption rate	Rahat-Rozenbloom et al., 2014
•Overweight (OWO) (42) •Lean (52)	3-d diet records, physical activity questionnaires, fecal samples	**OWO:** ↑ SCFA; dietary intakes and physical activity levels did not differ	Fernandes et al., 2014
•Indian individuals, 21–62 y (20): lean (5), normal (5), obese (5), surgically treated obese (5)	Fresh fecal samples, microbiota, and SCFA analysis	**Obese**: ↑ SCFA,↑*Bacteroides* **Treated-obese**: ↓SCFA ↓*Bacteroides*	Patil et al., 2012
•Advanced colorectal adenoma patients (A-CRA) (344) •Healthy control (344)	Dietary fiber intake, fecal SCFA, and microbiota analysis	**A-CRA group**: ↓SCFA production, ↓butyrate and butyrate-producing bacteria	Chen et al., 2013
•Celiac disease (CD) patients: normal diet, 13–60 y (10) and gluten-free, 21–66 y (11) •Healthy, 24–42 y (11)	Fresh fecal samples, microbiota, and SCFA analysis	**Untreated CD and treated CD**: ↑ SCFA than healthy **Treated CD patients**: ↓*Lactobacillus* and *Bifidobacterium* diversity	Nistal et al., 2012
**(B)**			
**Subjects, age (*****n*****)**	**Intervention diet (period)**	**Main outcomes**	**References**
•Healthy African Americans, 50–65 y (20) •Healthy South Africans, 50–65 y (20)	Own diet (2 w) followed by exchange to high-fiber, low-fat African-style (2 w) Own diet (2 w) followed by high-fat, low-fiber Western-style (2 w)	**African style diet**: ↑ butyrate; reciprocal changes in colon cancer risk biomarkers	O'keefe et al., 2015
•Healthy volunteers (23)	Cross-over: high red meat (HRM) diet vs. HRM plus butyrylated high-amylose maize starch (HAMSB) (4/4 w wash-out)	**HRM+HAMSB diet**:↑ excretion of SCFA and microbiota composition changes	Le Leu et al., 2015
•Healthy active volunteers (51)	Parallel-groups: butyrylated high amylose maize starch (HAMSB) vs. low-AMS (28 d)	**HAMSB diet**:↑free, bound and total butyrate and propionate	West et al., 2013
•Healthy volunteers, 20–50 y (17)	Cross-over: whole-grain (WG) vs. refined grain (2/5 w wash out)	**WG diet**: ↑acetate and butyrate	Ross et al., 2013
•Healthy volunteers, 18–85 y (63)	Cross-over: wheat bran extract (WBE) (3 or 10 g WBE) vs. placebo (0 g WBE; 3 w, 2 w wash-out)	**Daily intake of 10 g WBE**:↑bifidobacteria;↑ fecal SCFA and ↓ fecal pH	Francois et al., 2012
•Healthy volunteers, 18–24 y (60)	Parallel-groups: xylo-oligosaccharide (XOS) vs. inulin-XOS mixture (INU-XOS) vs. placebo (maltodextrin; 4 w)	**XOS**: ↑bifidobacteria and butyrate, and ↓acetate **INU-XOS**: ↑SCFA and propionate, and maintain acetate level	Lecerf et al., 2012
•Ulcerative colitis (UC) remission patients (19) •Healthy volunteers (10)	Cross-over: Australian diet vs. plus wheat bran-associated fiber and high amylose-associated resistant starch (8 w)	**Intervention diet**: did not correct the low gut fermentation in patients with UC	James et al., 2015
•Irritable bowel syndrome (IBS) with constipation woman, 20–69 y (32)	Parallel-groups: Milk acidified product (MP) vs. Fermented Milk product (FMP) (4 w)	**FMP**:↑potential butyrate producers, and ↑Total SCFA *in vitro*↑butyrate	Veiga et al., 2014
•IBS patients (27) •Healthy volunteers (6)	Cross-over: Australian diet vs. low FODMAP (Fermentable Oligo-, Di-, Mono-saccharides And Polyols) diet (21/21 d wash-out)	**Australian diet**:↑ relative abundance *Clostridium* cluster XIVa (butyrate-producer) **Low FODMAP diet**:↓total bacterial abundance	Halmos et al., 2015
•Cow's milk protein allergy infants (16) Healthy infants (12)	Cross-over:hydrolysed whey protein formula (eHF) without lactose vs. eHF containing 3.8% lactose (2 m)	**Addition of lactose**: ↑SCFA**;** ↑LAB and bifidobacteria; ↓*Bacteroides*/clostridia	Francavilla et al., 2012
•Obese women 18–65 y (30)	Parallel-groups: ITF vs. placebo (maltodextrin) (3m)	**ITF**:↓ total SCFA, acetate and propionate; ↑bifidobacteria	Salazar et al., 2015
•Obese men, 27–73 y (14)	Cross-over: high type III resistant starch (3 w) or high in wheat bran (3 w) and ended with weight-loss (low fat and carbohydrate, high protein, 3 w)	**Diet**: only explain 10% total variance in microbiota; amount of propionate correlated with *Bacteroidetes*	Salonen et al., 2014
•Obese volunteers, 45–77 y (6)	Cross-sectional: strict vegetarian diet (1 m)	↓SCFA;↓*Firmicutes/Bacteroidetes* ratio; ↑*Clostridium* clusters XIVa-IV; ↓*Enterobacteriaceae*	Kim et al., 2013
•Obese men, 21–74 y (17)	Cross-over: high-protein moderate-carbohydrate (HPMC) vs. high-protein low-carbohydrate (HPLC) (maintenance diet 7 d, 4 w)	**HPMC and HPLC diets**: ↑BSCFA (respect maintenance diet) **HPLC diet**: ↓butyrate and ↓*Roseburia/E.rectale*	Russell et al., 2011
•High Metabolic Syndrome risk volunteers (88)	Parallel-groups: High saturated fat (HS) vs. high monounsaturated fat (MUFA)/high glycaemic index (GI) (HM/HGI) vs. high MUFA/low GI (HM/LGI) vs. high carbohydrate (CHO)/high GI (HC/HGI) vs. and high CHO/low GI (HC/LGI) (24 w)	**High carbohydrate diets** (regardless GI):↑saccharolytic bacteria (including *Bacteroides* and *Bifidobacterium*) **High fat diets**:↓bacterial numbers **High saturated fat diet**:↑excretion of SCFA	Fava et al., 2013
•Hospitalized patients under enteral nutrition (41)	Parallel-groups: standard enteral formula vs. standard formula enriched FOS and fiber (12 d)	**FOS/fiber-enriched formula**: ↑butyrate	Majid et al., 2011

Although animal and human trials provide the best models for studying the influence of diet on the gut microbiota, *in vitro* fecal cultures constitute simpler approaches for investigating the interactions of diet and food components with the intestinal microbiota. Available *in vitro* models range from simple batch fermentation (Salazar et al., 2009; Arboleya et al., 2013b) to complex multi-stage continuous culture systems. The SHIME (Van Den Abbeele et al., 2010) and SIMGI models (Barroso et al., 2015) simulate the digestion from stomach to colon whereas the EnteroMix (Makivuokko et al., 2005) and the Lacroix models mimic the entire colonic process. TIM-2 reproduces the proximal colon and incorporates a dialysis membrane that simulates absorption of microbial metabolites by the body (Minekus et al., 1999). A microbial bias regarding butyrate and propionate producers occurs with some of these models (Van Den Abbeele et al., 2010), that could be alleviated by incorporating a simulation of the intestinal mucosa surface (Van Den Abbeele et al., 2013a). Labelling substrates with the stable isotope ^13^C makes possible to link the fermentation with specific members of the microbiota and to quantify production of metabolites (Maathuis et al., 2012) whilst the mathematical modeling is becoming a useful tool to study microbe-diet-host interactions (Shoaie et al., 2015).

When studying *in vitro* the influence of dietary components on microbial composition, the main aim usually is to increase beneficial bacteria and to enhance the production of SCFA whereas minimizing the synthesis of BSCFA. The fermentation of different substrates has been evaluated, ITF being the most studied (Sivieri et al., 2014). Starch (Fassler et al., 2006), arabinans, arabinoxylans (Van Den Abbeele et al., 2013b), galactooligosaccharides (Rodriguez-Colinas et al., 2013), xylitol (Makelainen et al., 2007), and lactulose (Cardelle-Cobas et al., 2009) have been also considered. The influence of polyphenols on the gut microbiota metabolism is currently receiving considerable attention (Valdés et al., 2015). Different microbial fermentation patterns can be obtained depending on physico-chemical characteristics of the substrates, speed of fermentation and the microbial populations involved in the process (initial breakdown of long polymers, direct fermentation of substrates, and cross-feeding interactions; Hernot et al., 2009; Zhou et al., 2013; Puertollano et al., 2014). Probiotics and their extracellular components (exopolysaccharides), can also act as modulators of SCFA microbial formation (Salazar et al., 2009; Van Zanten et al., 2012). In addition, a large number of studies highlight the influence of different foods and long-term diets on the intestinal microbiota activity and specifically, over the pattern of SCFA (Yang and Rose, 2014; Costabile et al., 2015).

The basal microbiota composition has also a profound influence on the final effects exerted *in vitro* by diet on microbial populations and metabolic activity (Arboleya et al., 2013a; Souza et al., 2014). In this regard, it has been found a different response to probiotics and prebiotics by the microbiota of individuals from different groups of age (Arboleya et al., 2013a; Likotrafiti et al., 2014), or between obese and lean people (Yang et al., 2013).

## Biological effects of SCFA

One of the health effects attributed to the production of SCFA is the concomitant reduction of the luminal pH, which by itself inhibits pathogenic microorganisms and increases the absorption of some nutrients (Macfarlane and Macfarlane, 2012). Acetate has been found to be a key player in the ability of bifidobacteria to inhibit enteropathogens (Fukuda et al., 2011). Moreover, butyrate fuels the intestinal epithelial cells and increases mucin production which may result in changes on bacterial adhesion (Jung et al., 2015) and improved tight-junctions integrity (Peng et al., 2009). Thus, the production of SCFA seems to play an important role in the maintenance of the gut barrier function.

After their production, SCFA will be absorbed and used in different biosynthetic routes by the host (Den Besten et al., 2013b). During the intestinal absorption process part of the SCFA, mainly butyrate, will be metabolized by the colonocytes (Pryde et al., 2002) whilst the rest will be transported by the hepatic vein and go into the liver, where they will be metabolized (Den Besten et al., 2013b). These SCFA will enter diverse CHO and lipid metabolic routes; propionate will mainly incorporate into gluconeogenesis whilst acetate and butyrate will be mostly introduced into the lipid biosynthesis. The involvement of SCFA in energy and lipid metabolism attracted the attention of researchers toward the potential role of SCFA in the control of metabolic syndrome. A reduction in obesity and insulin resistance in experimental animals on high-fat diet after dietary supplementation with butyrate has been observed (Gao et al., 2009). This protective effect of SCFA on the high-fat diet-induced metabolic alterations seems to be dependent on down-regulation of the peroxisome proliferator-activated receptor gamma (PPARγ), therefore promoting a change from lipid synthesis to lipids oxidation (Den Besten et al., 2015). Interestingly although the three main intestinal SCFA have a protective effect on diet-induced obesity, butyrate and propionate seem to exert larger effects than acetate (Lin et al., 2012). Different mechanisms have been proposed to explain these effects, the activation of signaling pathways mediated by protein kinases, such as AMP-activated protein kinase (Gao et al., 2009; Peng et al., 2009; Den Besten et al., 2015) or mitogen-activated protein kinases (MAPK; Jung et al., 2015), being a common observation. Butyrate and propionate, but not acetate, have been reported to induce the production of gut hormones, thus reducing food intake (Lin et al., 2012). Acetate has also been found to reduce the appetite, in this case through the interaction with the central nervous system (Frost et al., 2014). However, in spite of these promising animal data, controlled human intervention studies are still needed before drawing firm conclusions (Canfora et al., 2015).

It has also been observed that SCFA protect against the development of colorectal cancer (CRC), with most studies focusing on butyrate (Canani et al., 2011; Keku et al., 2015). Butyrate promotes colon motility, reduces inflammation, increases visceral irrigation, induces apoptosis, and inhibits tumor cell progression (Zhang et al., 2010; Canani et al., 2011; Leonel and Alvarez-Leite, 2012; Keku et al., 2015), all of these properties being beneficial in CRC prevention. In cancerous colonocytes, due to the Warburg effect, butyrate accumulates, which increases its activity as inhibitor of histone deacetylation, promoting apoptosis of CRC cells. Interestingly, a recent animal study suggests that the protective effect of dietary fiber upon CRC is dependent on the production of butyrate by the microbiota (Donohoe et al., 2014).

In addition, butyrate and propionate have also been reported to induce the differentiation of T-regulatory cells, assisting to control intestinal inflammation; this effect seems to be mediated via inhibition of histone deacetylation (Donohoe et al., 2014; Louis et al., 2014). This control of intestinal inflammation may result beneficial in terms of gut barrier maintenance, reducing the risk of inflammatory bowel disease or CRC. Unlike what happens with the three main intestinal SCFA, acetate, propionate, and butyrate, little is known about the potential health effects of other SCFA.

## Concluding remarks

The main role of diet is to provide enough macro- and micronutrients to fulfill daily requirements and well-being. However, during the last decades the association between dietary intake and physiology has been increasingly-recognized, although many of the molecular and immunological aspects by which dietary components could influence human health remain still largely unknown. Bacterial fermentation of CHO and proteins produces SCFA which emerge as major mediators in linking nutrition, gut microbiota, physiology and pathology. Many biological effects seem to be mediated by these bacterial metabolites but a conclusive proof is not available for many of the health claims made for SCFA. Promising *in vitro* and animal studies have been published but they cannot be easily extrapolated to the human situation. The design of improved approaches combining *in vitro, in vivo*, and “omics” technologies should be carried out, with emphasis in human intervention trials, to explore the mechanisms of production and action of SCFA, thus opening the possibility to find strategies for developing personalized nutrition.

## Author contributions

All authors listed, have made substantial, direct and intellectual contribution to the work, and approved it for publication.

### Conflict of interest statement

The authors declare that the research was conducted in the absence of any commercial or financial relationships that could be construed as a potential conflict of interest. The reviewer BJ and handling Editor declared their shared affiliation, and the handling Editor states that the process nevertheless met the standards of a fair and objective review
